# Medical and Surgical Nursing

**Published:** 1901-01

**Authors:** 


MEDICAL AND SURGICAL NURSING. A treatise on modern nursing from the physician's and surgeon's standpoint, for the guidance of graduate and student nurses, together with practical instruction in the art of cooking for the sick. Edited by H. J. O'BRIEN, M. D., Professor of Clinical Surgery, Hamline University; Surgeon to St. Joseph's Hospital, St. Paul, etc. Duodecimo, pp. ix. to 287. New York: G. P. Putnam's Sons. 1900. [Price, $1.50.]

There are plenty of books on nursing abroad in the land. To gain a place for itself a new book must have especial merit. This work makes a strong claim for favor in the very plan of it. It contains fifteen chapters, each, with one exception, written by a physician who presents his subject as he believes the nurse should understand it. The chapter headings read as follows: General nursing, bacteria; asepsis and antisepsis; anesthesia; shock; deformities; acute infections and contagious diseases; fractures; dislocations, and wounds; diseases of women; obstetrics; infant and child; nervous and mental diseases; poisons; eye and ear; cooking for the sick. The last chapter is by a woman no less a specialist in the subject assigned her than are the other writers in theirs.

No one knows so well as the physician the things which it is desirable that the nurse should learn. Those who are seeking for a thoroughly good book to place in the hands of the pupil nurse to supplement and fix in her mind the lessons of the class-room, will do well to carefully examine this volume. We believe it fills a gap in the literature of nursing. M. J. F.

